# Integrating mental health care into primary care systems in low- and middle-income countries: lessons from PRIME and AFFIRM

**DOI:** 10.1017/gmh.2017.3

**Published:** 2017-04-18

**Authors:** T. Davies, C. Lund

**Affiliations:** 1 Department of Psychiatry and Mental Health, Alan J Flisher Centre for Public Mental Health, University of Cape Town, South Africa; 2 Centre for Global Mental Health, Institute of Psychiatry, Psychology and Neuroscience, King's College London, UK

**Keywords:** innovations, mental health, Policy and Systems, PRIME, AFFIRM, task sharing

## Introduction

Mental, neurological and substance use (MNS) disorders are now the leading cause of disability worldwide, contributing to 23% of global years of life lived with disability (Whiteford *et al.* 2013). Most of this burden is carried by low- and middle-income countries (LMICs). However, MNS disorders in LMICs do not receive the research attention or service personnel they deserve (Saxena *et al.* 2006), with the demand for services for outweighing the provision of services and treatment personnel. This gap in service provision is often referred to as the ‘treatment gap’.

In response, a number of research priorities were proposed for MNS disorders by the Grand Challenges in Global Mental Health initiative in an article published in *Nature* in 2011 (Collins *et al.* 2011). In keeping with this agenda, there has been a burgeoning of new innovations in global mental health in the last 5–6 years, including a wide range of initiatives funded by the US National Institute of Mental Health (NIMH), the European Commission (EC), the UK Department for International Development (DFID), Grand Challenges Canada and the Wellcome Trust, among others.

These innovations aim to provide a concrete evidence base to test and demonstrate the effectiveness of interventions to narrow the treatment gap. One key strategy is to integrate mental health into primary care systems in LMICs, through task shifting or task sharing, in line with the objectives of the WHO global mental health action plan (WHO,^22,23^).

This paper will present case studies of two such initiatives: the PRogramme for Improving Mental health carE (PRIME) and the Africa Focus on Intervention Research for Mental health (AFFIRM). We will then draw out lessons from our experiences of integrating mental health into primary care in LMICs.

## PRIME

PRIME is a consortium of research institutions working collaboratively with Ministries of Health in five countries (Ethiopia, India, Nepal, South Africa and Uganda), led from the University of Cape Town (UCT) with partners in the UK and the WHO, and funded by DFID (Lund *et al.* 2012). The purpose of PRIME is to generate high-quality research evidence on the implementation and scaling up of treatment programmes for priority mental disorders in primary and maternal health care contexts in low resource settings, by adapting, implementing and evaluating the WHO's mental health Gap Action Programme Intervention Guide (mhGAP-IG) (WHO, 2010). The mhGAP-IG was developed as a manual that provides guidelines for health care practitioners to deliver mental health services at the primary health care level, through a system of skill-transfer and support from specialists. The types of health care practitioners functioning as non-specialist mental health workers (NSWs) vary across PRIME sites, and include community health workers, nurses, health extension workers, village health workers, medical officers and HIV counsellors.

PRIME works closely with Ministries of Health, health care providers, academic institutions and civil society organisations to set up ‘district demonstration sites’ in each of the five countries. PRIME has worked in each site with the stakeholders to develop site-specific district ‘Mental health care plans’ (MHCP), based on the mhGAP-IG (Lund *et al.* 2016). It is now testing the feasibility, acceptability, cost and impact of these plans in each country site, and moving to scale up the models in other districts.

By generating research evidence of integrating mental health care into primary and maternal health systems, PRIME aims to make a direct contribution to reducing the treatment gap, not only in the five PRIME countries, but also in other low resource settings. PRIME also promotes sustainable research capacity in the participating country institutions to develop, undertake and disseminate the research being conducted.

There are numerous challenges that PRIME is facing in implementing task-shared treatment programmes at the primary care level. These include financial constraints, low levels of community awareness and high levels of stigma, the absence of supportive health system structures, poor information about treatment coverage, and a shortage of mental health care specialists to supervise and support NSWs. For each of these challenges, a number of strategies have been adopted, using the ‘Theory of Change’ (ToC) as an overall framework (Breuer *et al.* 2016). The strategies are elaborated in the ‘lessons’ section, below.

## AFFIRM

AFFIRM is a research and capacity building hub investigating task sharing of mental health care in low-income settings in Africa (Lund *et al.* 2015). It is conducting two Randomised Controlled Trials (RCTs) in South Africa and Ethiopia, and capacity building programmes across six countries in sub-Saharan Africa. It is also involved in a shared project with the other NIMH research hubs. This project is examining the barriers and facilitators to scaling up task sharing in LMICs, and developing tools to assess these barriers and facilitators. AFFIRM is coordinated through UCT, with academic centres in the US and the UK. It is one of five collaborative ‘Hubs’, funded by the NIMH, focused on global research in mental health.

The RCT in South Africa aims to determine the cost-effectiveness of a task-shared counselling intervention for maternal depression, using NSWs, compared with enhanced usual care in Khayelitsha, South Africa. The objective of the RCT in Ethiopia is to determine the cost-effectiveness of task-shared care for persons with severe mental disorders using NSWs, compared with a model of specialist mental health care in Ethiopia.

AFFIRM also aims to build individual and institutional capacity for intervention research in mental health in sub-Saharan Africa. This is addressed through an MPhil programme in Public Mental Health at UCT and Stellenbosch University, support for PhD students linked to the projects, and provision of short courses covering topics such as RCTs and operational research for mental health.

## Lessons for integrating mental health into primary care in LMICs

Experiences from PRIME and AFFIRM provide several potential lessons for policy makers, researchers and practitioners interested in integrating mental health into primary care systems in LMICs.

### Engage actively and collaboratively with local stakeholders

When designing and planning local services, it is important to do so using participatory methods such as ToC workshops. These workshops proved to be a useful approach to developing a logical structure for mental health plans in PRIME, through accessing contextual details for implementing the plans in district sites and obtaining stakeholder buy-in (Breuer *et al.* 2014). The participatory nature of the workshops allows for an exploration of the suitability of locally based services, and involves all stakeholders working together to map out the ToC for the district, while keeping the intended outcomes or end goals in mind. To ensure inclusion and maximum buy-in, we recommend including a range of stakeholders, such as ‘mental health specialists, researchers, policy makers, district level health planners and management, and service providers’ (Breuer *et al.* 2014, p. 5). Also, when conducting ToC workshops, it is important to keep the hierarchical nature of health systems in view. We found that some contexts may require separate workshops to involve different levels of participants, in order to facilitate maximum participation, and high-level stakeholders may potentially require separate interviews. Buy-in is only achieved with consultation with all stakeholders, through both ‘a top down and bottom up approach’ (Breuer *et al.* 2014, p. 10).

### Use primary care systems to access vulnerable populations

Improving access to mental health care for vulnerable populations can often be achieved through primary health care centres. For example, in South Africa, there is a very good uptake of antenatal service attendance at primary care clinics: 93% of pregnant women accessed these services in 2014 (StatisticsSA., 2015). In addition, previous epidemiological studies have identified high prevalence of antenatal and postnatal depression and anxiety in this population, in Khayelitsha (Cooper *et al.* 2009). The AFFIRM South Africa trial took this opportunity to access depressed pregnant women in antenatal clinics. This carries the potential to improve maternal and child health outcomes, in keeping with the priorities of the sustainable development goals. We found that while working in primary care systems, it is essential to achieve buy-in from local providers such as nurses. Meetings, individual discussions and networking with health care providers before implementation are vital for an intervention to be effective.

### Use cultural concepts of distress and narrative-based vignettes to identify persons with potential mental health problems

The PRIME site in Nepal developed a Community Informant Detection Tool (CIDT) for detecting mental health problems by NSWs. This tool incorporates locally identified cultural concepts of distress and manifestations of mental health problems that are commonly understandable, and uses vignettes rather than a checklist of symptoms, with good accuracy (Jordans *et al.* 2015). The Nepali PHQ-9 also incorporated cultural concepts of distress to improve accuracy and reduce the screening burden on NSWs using a stepped algorithm approach. In AFFIRM, local concepts of maternal depression were identified through in-depth interviews with participants and these were incorporated into the development of the AFFIRM counselling manual to ensure its local relevance and acceptability (Davies *et al.* 2016; Nyatsanza *et al.* 2016).

### Use manual-based approaches to deliver care through non-specialist health workers

There is now a reasonably well-established evidence base for using manuals to guide non-specialist counsellors in the delivery of psychological interventions (Rahman *et al.* 2008; Chibanda *et al.* 2011; Balaji *et al.* 2012). Both PRIME and AFFIRM are using manualised counselling interventions for perinatal depression, general adult depression and alcohol use disorders, which provide a systematic and structured method of working with these illnesses for NSWs. Piloting the manual-based interventions, and evaluating them for understanding and comprehension, is essential, before rolling them out with NSWs.

### Set up systems of ongoing supervision and support

Experiences from PRIME and AFFIRM have shown that in order for programmes to be sustainable and effective it is critical for NSWs to have ongoing, structured and supportive supervision. We found that supervision needs to be conducted by specialist mental health workers, and not by non-specialists in existing supervisory structures, unless they have been specifically trained. This however requires the availability of specialists who can provide the necessary support and referral platforms, in order for NSWs to be able to implement the required mental health services (Mendenhall *et al.* 2014). Training in itself is not enough to provide task-shared services by NSWs if there are not sufficient specialists available to provide supervision and referral platforms for severe cases.

In the AFFIRM-SA trial, we found weekly group and monthly individual supervision by a mental health specialist were essential for NSWs to competently treat women with maternal depression with manual-based counselling sessions. We found that 5 days of training was insufficient to obtain competence in counselling for community health workers. With hindsight, more extensive training, and more in-depth supervision would have further improved counsellors’ competence. We thus recommend supervision to occur weekly if in groups, or bi-monthly or monthly if individualised. PRIME has developed a rating scale which was used in Nepal to monitor training and supervision, which can be administered across programme types [The ENhancing Assessment of Common Therapeutic factors (ENACT) rating scale] (Kohrt *et al.* 2015), as well as a self-report competency evaluation form to monitor NSWs competency post training. These and other tools such as counselling manuals are available on the PRIME website (www.prime.uct.ac.za).

Along with supervision, it is necessary to monitor progress against the programme aims, throughout implementation, to (a) ensure implementation is being carried out according to protocol; and (b) ensure the intervention is continuously appropriate, beneficial and relevant to the local setting. This can be conducted by having clearly stated monitoring methods and goals written in project protocols, which can guide those monitoring progress and data. AFFIRM's mental health specialist was responsible for monitoring number and quality of sessions provided by NSWs, while also listening to recorded sessions in order to monitor quality. PRIME monitors progress and data through ongoing multi-method evaluation strategies across different levels, which examine changes in contact rates, detection of disorders, patient outcomes and the process of implementation (De Silva *et al.* 2016).

### Adequately compensate NSWs for their services

In PRIME, the NSWs across sites stated the need for adequate compensation in order to add mental health care to their work load within the primary care systems. Lack of adequate compensation can be a barrier to continuation of services and commitment by health workers because of their already heavy workloads. PRIME involves varying already-established NSWs, who receive compensation as part of their employment in existing routine Ministry of Health or non-governmental (NGO) services. What is slightly more difficult to sustain is compensation of higher level supervisors or specialists, some of whom have had to be funded by PRIME in order to demonstrate how the model could work in routine settings. We are currently working with Ministries of Health and local NGOs to take over these additional functionalities in a sustainable manner, beyond the life of the programme.

### Respond to crises by ‘building back better’

When primary care mental health systems are strengthened, more resources are available to respond to local crises. The WHO report on mental health in humanitarian disasters recommends using these opportunities to ‘build back better’ out of a disaster (WHO, 2013*a*). The PRIME site in Nepal provided an example of this when it was able to respond to the crisis of the earthquake in 2015 by making PRIME Mental health care plans available to assist with strengthening mental health services in earthquake affected districts. These included psychosocial skills modules, counselling manuals, detection tools, and mhGAP implementation plans.

### Make use of policy windows

Lastly, we recommend making use of ‘policy windows’, by providing solutions or recommendations for problems that have been identified and acknowledged by government at the time when they are open to finding solutions; in other words when there is political will or a desire for change. For example, PRIME took the opportunity to implement their mental health care plan in the Dr. Kenneth Kaunda district in South Africa, which was a national Department of Health (DoH) pilot site for the planned National Health Insurance reforms. In the case of the AFFIRM trial in South Africa, the Western Cape province DoH has launched a ‘First 1000 days initiative’, which draws attention to the importance of the first 1000 days of a child's life (DoH, 2016). This initiative focuses largely on maternal and child health and education, and provides an optimal policy window to use the AFFIRM trial findings to promote and initiate maternal mental health interventions in routine maternal health services. Similarly, in Ethiopia, the Ministry of Health developed a strategy to implement and scale up the mhGAP, which provided the opportunity for PRIME to present and use their tools in Ethiopia. In India, the PRIME mental health care plans were linked to a governmental initiative to expand access to mental health care in the state of Madhya Pradesh: the Strategy for Healthy and Active Minds (SOHAM) initiative.

Crucial to integrating mental health services into primary care is financing. AFFIRM was an externally funded RCT, and while it funded independent counsellors not employed by government, the purpose of the research was to demonstrate the cost-effectiveness of a task sharing model to policy makers, in the hope that they will invest in and scale up the interventions. PRIME on the other hand, worked closely with Ministry of Health partners, and relied largely on Ministry of Health financing to implement the interventions with training, technical support and supervision provided by PRIME. However, in some settings, where services were not in place, PRIME provided additional funding to initiate these services, in order to demonstrate the feasibility and impact of new models. PRIME is now working with Ministries of Health and NGOs to ensure sustainability and scale up of these interventions by local funding sources, including Ministries of Health and NGOs.

## Conclusion

The burden of MNS disorders is substantial, and task sharing innovations in primary care have been recommended to address this burden in LMICs. PRIME and AFFIRM are two innovations attempting to address the treatment gap for MNS disorders in these settings. Although results of the impact of these initiatives are not yet available, the above lessons are based on our experiences of the process of delivery, and close collaboration with a range of stakeholders in Ministries of Health and NGOs. Careful attention to the detail in addressing the ‘how’ questions of implementation and scale-up are essential if vulnerable populations are to be reached.
